# Cancer-related ectopic expression of the bone-related transcription factor RUNX2 in non-osseous metastatic tumor cells is linked to cell proliferation and motility

**DOI:** 10.1186/bcr2762

**Published:** 2010-10-28

**Authors:** David T Leong, Joleen Lim, Xuewei Goh, Jitesh Pratap, Barry P Pereira, Hui Si Kwok, Saminathan Suresh Nathan, Jason R Dobson, Jane B Lian, Yoshiaki Ito, P Mathijs Voorhoeve, Gary S Stein, Manuel Salto-Tellez, Simon M Cool, Andre J van Wijnen

**Affiliations:** 1 Cancer Science Institute of Singapore, National University of Singapore, 28 Medical Drive, 117456 Singapore; 2 Department of Chemical and Biomolecular Engineering, National University of Singapore, Block E5- 02-09, 4 Engineering Drive 4, 117576 Singapore; 3 Department of Cell Biology and Cancer Center, 55 Lake Avenue North, University of Massachusetts Medical School, Worcester, MA 01655, USA; 4 Current address: Department of Anatomy and Cell Biology, Rush University Medical Center, 600 S. Paulina Street, Chicago, IL 60612, USA; 5 Musculoskeletal Research Laboratories, Department of Orthopaedic Surgery, National University of Singapore, 1E Kent Ridge Road, NUHS Tower Block-Level 11, 119228 Singapore; 6 Division of Musculoskeletal Oncology, Department of Orthopaedic Surgery, Yong Loo Lin School of Medicine, National University of Singapore, 5 Lower Kent Ridge Road, 119074 Singapore; 7 Cancer and Stem Cell Biology Program, Duke-National University of Singapore Graduate Medical School, Khoo Teck Puat Building Level 7, 8 College Road, 169857 Singapore; 8 Department of Biochemistry, Yong Loo Lin School of Medicine, National University of Singapore, 8 Medical Drive, 117597 Singapore; 9 Department of Pathology, National University Health System, National University of Singapore, 5 Lower Kent Ridge Road, 119074 Singapore; 10 Stem Cells and Tissue Repair, Institute of Medical Biology, A*STAR, 8A Biomedical Grove, #06-06 Immunos, 138648 Singapore

## Abstract

**Introduction:**

Metastatic breast cancer cells frequently and ectopically express the transcription factor RUNX2, which normally attenuates proliferation and promotes maturation of osteoblasts. RUNX2 expression is inversely regulated with respect to cell growth in osteoblasts and deregulated in osteosarcoma cells.

**Methods:**

Here, we addressed whether the functional relationship between cell growth and *RUNX2 *gene expression is maintained in breast cancer cells. We also investigated whether the aberrant expression of RUNX2 is linked to phenotypic parameters that could provide a selective advantage to cells during breast cancer progression.

**Results:**

We find that, similar to its regulation in osteoblasts, RUNX2 expression in MDA-MB-231 breast adenocarcinoma cells is enhanced upon growth factor deprivation, as well as upon deactivation of the mitogen-dependent MEK-Erk pathway or EGFR signaling. Reduction of RUNX2 levels by RNAi has only marginal effects on cell growth and expression of proliferation markers in MDA-MB-231 breast cancer cells. Thus, RUNX2 is not a critical regulator of cell proliferation in this cell type. However, siRNA depletion of RUNX2 in MDA-MB-231 cells reduces cell motility, while forced exogenous expression of RUNX2 in MCF7 cells increases cell motility.

**Conclusions:**

Our results support the emerging concept that the osteogenic transcription factor RUNX2 functions as a metastasis-related oncoprotein in non-osseous cancer cells.

## Introduction

Runt-related (Runx) transcription factors [1] are lineage-specific developmental regulators and defects in their regulatory functions have been pathologically linked to a broad spectrum of cancers [2-7]. Normal endogenous expression of Runx proteins is biologically linked to cell growth suppression. Consistent with this growth suppressive role, Runx proteins are functionally inactivated or altered in distinct cancer types [2-7]. Yet, elevated or ectopic expression of Runx proteins may contribute to the tumorigenic and/or metastatic properties of cancer cells [2-7]. These findings together suggest that Runx proteins can function as bona fide tumor suppressors or classical oncoproteins depending on the cellular context. Current evidence indicates that RUNX2 is a key pathological factor in metastatic breast [8-17], prostate [18-22] and bone [23-31] cancer cells, as well as in lymphomas in mouse models [32-35]. To understand the oncogenic contribution of RUNX2 to the etiology of these diverse cancers, it is necessary to define the pathological mechanisms by which RUNX2 perturbs cellular physiology.

During normal development, RUNX2 is a principal component of a genetic regulatory pathway that controls osteoblast maturation and bone formation *in vivo *[36-40]. Importantly, loss of RUNX2 function deregulates osteoblast proliferation *ex vivo *[23,41-43], while experimental elevation of RUNX2 protein levels suppresses proliferation in different osteogenic mesenchymal cell types [23,41,44]. RUNX2 activity is functionally coupled with the osteoblast cell cycle and elevated in quiescent cells [23,41]. RUNX2 levels are selectively up regulated after mitosis during early G1 by both transcriptional and post-transcriptional mechanisms and down regulated prior to entry in S phase to avoid a cell growth delay in normal osteoblasts [23,45-47]. Taken together, these findings indicate that RUNX2 functions as a cell growth suppressor in primary diploid osteoblasts where the protein is endogenously expressed. However, RUNX2 destabilization is compromised in several osteosarcoma cell types that express constitutively high levels of RUNX2 [23-26], suggesting that bone cancer cells may bypass the growth suppressive properties of RUNX2.

RUNX2 performs proliferation-related functions in osteoblasts that may be linked to its biological activities in human cancers. For example, RUNX2 loss of function blocks senescence, as reflected by a loss of p19ARF expression, loss of chromosomal integrity and delayed DNA repair [42,43]. RUNX2 also functions as an epigenetic regulator that controls osteoblast growth by attenuating ribosomal gene expression and protein synthesis [48,49]. Gene expression profiling and gene ontology analysis of RUNX2 responsive programs revealed that RUNX2 regulates genes involved in G protein coupled receptor signaling [44], sterol/steroid metabolism [50], RNA processing [51] and proteoglycan synthesis [52]. Several of the encoded proteins have pro-mitogenic or pro-survival functions in osteoprogenitors, including the estrogen-responsive G protein coupled receptor GPR30 and its downstream regulator RGS2, as well as Cyp11a1, which produces the steroid precursor pregnenolone [44,50]. Thus, these RUNX2 target genes may contribute to the oncogenic activity of RUNX2 in osseous or non-osseous tumors.

Our understanding of the role of RUNX2 in osteoblasts and osteosarcoma cells where the gene is endogenously expressed [23-29], provides a biological framework for analyzing the regulation and regulatory roles of RUNX2 in non-osseous cancer cells (for example, breast) in which RUNX2 is ectopically expressed [8-17]. Prior studies indicate that RUNX2 is required for osteolytic lesions of either breast cancer or prostate cancer cells upon intra-tibial injection and cell culture models indicate that RUNX2 expression stimulates cell invasion [8,11,12,21]. In this study, we examined how RUNX2 levels are modulated with respect to cell growth, as well as whether RUNX2 controls the metastatic properties of breast cancer cells in culture. The main finding is that RUNX2 is required for cell motility of breast cancer cells. Furthermore, RUNX2 levels are elevated upon cell growth inhibition in breast cancer cells, but cell growth is only marginally enhanced upon RUNX2 depletion by RNA interference. Our studies support the general concept derived from multiple studies that RUNX2 may function as a metastasis-related oncoprotein in non-osseous cancer cells.

## Materials and methods

### Cell culture, proliferation assays and inhibitors treatment

Human MDA-MB-231 and MCF-7 breast cancer cell lines were cultured in Dulbecco's modified Eagle's medium (DMEM, Gibco, Carlsbad, CA, USA) supplemented with 10% fetal bovine serum (FBS, Hyclone, Waltham, MA, USA), 5% L-glutamine (PAA, Pasching, Austria) and 1% penicillin/streptomycin (PAA, Pasching, Austria) at 37°C and 5% CO_2_. Cell proliferation was measured by performing live cell counts in triplicate using Trypan Blue exclusion as a measure for cell viability. Inhibition of MAPK dependent signaling pathways was carried out by treatment with the MEK1 inhibitor PD98059 (#9900, Cell Signaling Technology, Inc., Beverly, MA, USA). The inhibitor was prepared as a 10 mM stock solution in dimethyl sulfoxide (DMSO). MDA-MB-231 and MCF-7 cells were plated at a density of 3 × 10^5 ^cells per well in six-well plates and incubated overnight. Cells were then treated with various concentrations of PD98059 (that is, 0, 1, 5, 10, 20 and 50 μM) and incubated for two hours at 37°C before preparation of whole cell lysates. Stock cycloheximide was dissolved in DMSO at 100 mM concentration and freshly added into the media.

### Western blot analysis

Lysates were prepared from cells washed with ice-cold phosphate-buffered saline (PBS, pH 7.2), scraped and lysed into 100 μl of 1 × SDS-PAGE protein loading buffer (31.25 mM Tris-HCl, pH 6.8, 12.5% glycerol, 2.5% β-mercaptoethanol, 1% sodium dodecyl sulfate (SDS), 0.005% bromophenol blue and 1% Roche complete protease inhibitors cocktail). The cell suspension was sonicated for a few seconds to disperse the cells and cell debris was pelleted by centrifugation at 14,000 g for 10 minutes at 4°C. The supernatant containing the protein fraction was collected and boiled for five minutes. Protein samples were then stored at -20°C until further analysis.

Equal amounts of protein from each treatment group were resolved on a 10% SDS-PAGE gel (100 V, 120 minutes) and subsequently transferred onto a nitrocellulose membrane (100 V, 60 minutes). Blots were blocked with 5% nonfat dried milk in PBS-0.1% Tween 20 (PBS-T) solution for 30 minutes prior to primary antibody incubation overnight at 4°C. Primary antibodies to proteins of interest were diluted in PBS-T solution containing 3% bovine serum albumin (BSA) at a ratio of 1:1,000. After incubation with primary antibodies, blots were washed three times for five minutes each with PBS-T solution and incubated for one hour at room temperature with the appropriate secondary antibody at a 1:5,000 dilution in PBS-T solution containing 5% nonfat dried milk. Following incubation, blots were washed three times for five minutes each with PBS-T solution, and the antibody binding was detected with SuperSignal West Pico Chemiluminescent substrate (Thermo Scientific, Waltham, MA, USA) by exposing blots to XAR-5 film (Kodak, Rochester, NY, USA).

The primary antibodies used were RUNX2 mouse monoclonal antibody (D130-3, MBL International, Woburn, MA, USA), phospho-p44/42 MAP kinase E10 mouse monoclonal antibody (#9106, Cell Signaling Technology, Inc, Beverly, MA, USA), p44/42 MAP kinase (ERK1/2) rabbit polyclonal antibody (#9102, Cell Signaling Technology, Inc), p21 rabbit polyclonal antibody (sc-397, Santa Cruz Biotechnology, Santa Cruz, CA, USA), p53 rabbit polyclonal antibody (sc-6243, Santa Cruz), c-myc mouse monoclonal antibody (sc-40, Santa Cruz) and GAPDH mouse monoclonal antibody (sc-32233, Santa Cruz). The secondary antibodies used were horseradish peroxidase (HRP)-conjugated goat anti-mouse IgG antibody (sc-2005, Santa Cruz) and HRP-conjugated goat anti-rabbit IgG antibody (sc-2004, Santa Cruz).

### Real-time quantitative Reverse Transcriptase PCR (qRT-PCR)

Total RNA was prepared according to manufacturer's instructions (Qiagen RNeasy Mini kit, Qiagen, Hilden, Germany). In-column genomic DNA digestions were performed using DNase I (Qiagen). Total RNA collected samples were quantified and reversed-transcribed to cDNA and 5 ng of cDNA was amplified on the ABI 7300 Real-Time PCR System using fluorescent SYBR Green PCR master mix (Fermentas, Thermo Scientific, Waltham, MA, USA) with the following sets of primers: *p21 *(forward primer: 5'-GTCCGTCAGAACCCATGC-3', reverse primer: 5'-GTCGAAGTTCCATCGCTCA-3'), *cyclin D1 *(forward primer: 5'-TGAACAAGCTCAAGTGGAACC-3', reverse primer: 5'-GTTTGCGGATGATCTGTTTGT-3') and *GAPDH *(forward primer: 5'-GAGTCCACTGGCGTCTTCA-3', reverse primer: 5'-GTTCACACCCATGACGAACA-3'). Average fold changes were calculated by differences in threshold cycles (Ct) between pairs of samples. *GAPDH *gene was used as an internal control.

### Retrovirus packaging and transduction

A retrovirus plasmids based on pSUPER-Retro (Oligoengine, Seattle, WA, USA) was generated that encode short hairpins against *RUNX2*. The pSUPER-Retro-shRUNX2 construct and the empty control plasmid were each transfected into ecotropic virus packaging cells (Ecopacks; Clontech, Mountain View, CA, USA) using the calcium phosphate precipitation method. Retroviral supernatant was collected at 48 h after transfection, rapidly frozen in liquid nitrogen and stored at -80°C. During transduction, retroviral supernatants were mixed with culture media at a 1:1 ratio with polybrene (8 μg/ml) and added to cells and incubated overnight. Cells containing the retroviral plasmids were recovered by antibiotic selection for five days using puromycin (2.5 μg/ml).

### Cell migration assays

Wound healing assays were used to measure cell migration. Cells in various groups were seeded in six-well plates at 5 × 10^5 ^cells per well to form a confluent layer of cells overnight. A line of cells was mechanically removed by scratching the cell layer with a 200 μl pipette tip to create a 'wound'. Cells migrating into the 'wound' area were monitored at 30 minutes intervals by automated image collection at the same wound location with a Nikon Eclipse Live Cell Imaging system. Three separate regions along the wound were randomly chosen as scratch zones. The data were analyzed using the accompanying Nikon NIS-Elements software provided by the manufacturer (Nikon Corporation, Chiyoda-ku, Tokyo, Japan). During image collection, cells were maintained under sterile culture conditions at 37°C in an atmosphere containing 5% CO_2_.

### Statistical analysis

Analysis of co-variance (ANCOVA) tests (PASW version 17, SPSS Inc., Chicago, IL, USA) were used to statistically assess the significance of differences between either siRUNX2 group or RUNX2 overexpression group vs control in the time course migration assays. The level of significance was set at *P *< 0.05.

## Results

### Expression of RUNX2 in breast cancer cells is enhanced upon cessation of cell growth

**Figure 1:**
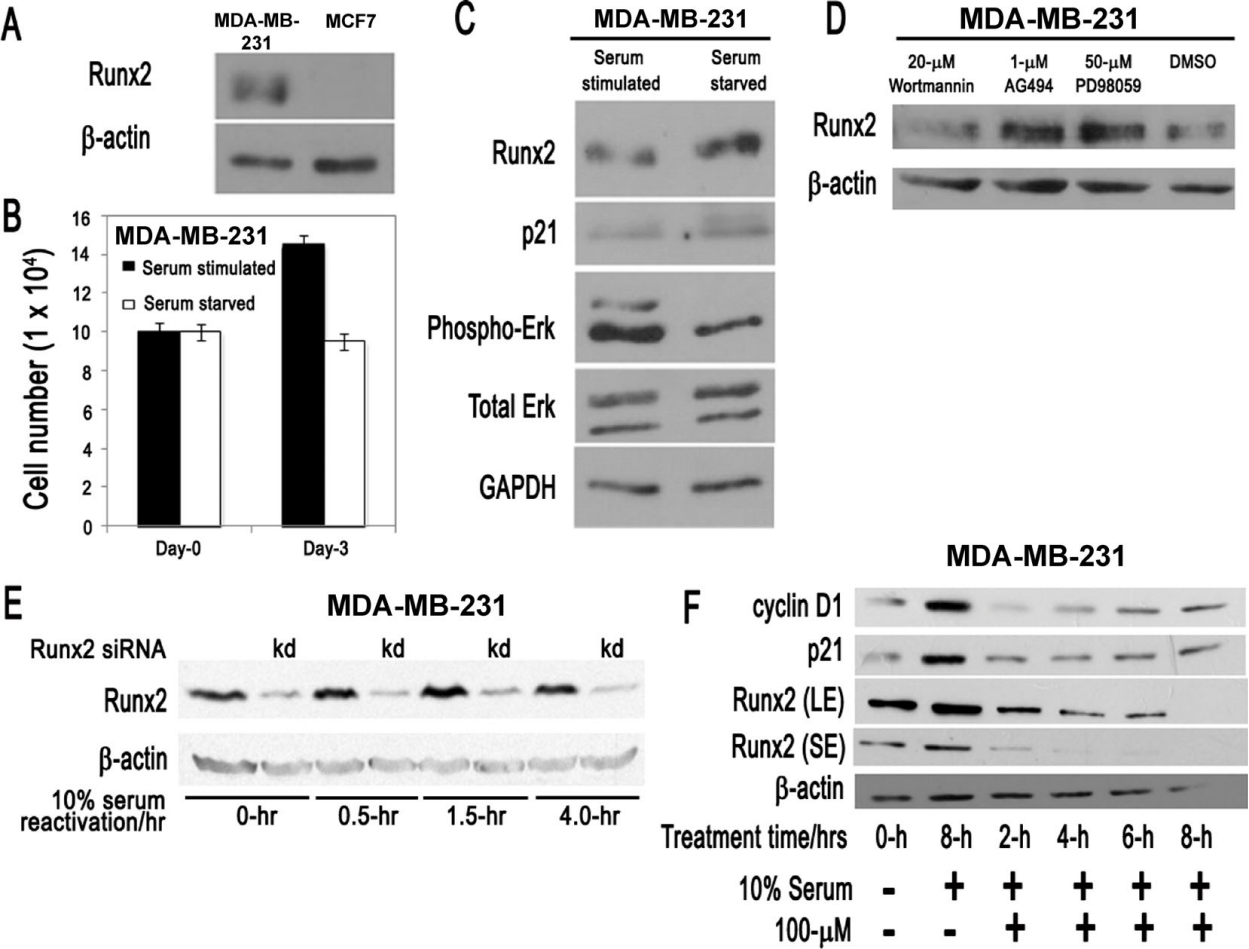
**RUNX2 has an anti-mitogenic function in MDA-MB-231 cells**. **(A) **Western blot analysis shows that RUNX2 protein expression is evident in MDA-MB-231 cells but below the level of detection in MCF7 cells. **(B) **Serum stimulation of MDA-MB-231 cells enhances cell proliferation as reflected by cell counts of cultures supplemented with and without serum (on Days 0 and 3). Cells were plated, serum starved for 48 h and then released for 24 h with and without 10% serum. Live cell counts were performed in triplicate using trypan blue dye-exclusion. **(C) **Serum starvation of MDA-MB-231 cells increases RUNX2 and p21 protein levels. MDA-MB-231 cells were supplemented with (lane 1) and without (lane 2) serum. The protein expression levels of RUNX2, p21, ERK1/2 and phospho-ERK were analyzed by western blotting. ERK1/2 antibody was used to detect the endogenous level of total-ERK protein in the cells. GAPDH provided a control for equal loading of lysates. **(D) **The MEK inhibitor PD98059 and the epidermal growth factor receptor (EGFR) kinase inhibitor each significantly increase the expression of RUNX2 in MDA-MB-231 cells by western blotting; β-actin was used as a control for protein loading. The phosphoinositide 3-kinase (PI3K) inhibitor Wortmannin has no effects on RUNX2 levels. MDA-MB-231 cells were incubated in complete medium for 2 h with each of the inhibitors. **(E) **RUNX2 remains stable for 4 h after serum re-activation. MDA-MB-231 cells were serum starved overnight and serum stimulated for the indicated lengths of time (in hours). Cells were also transfected in parallel with an empty vector (odd lanes) or a viral vector expressing shRUNX2 (even lanes) that 'knock down' (kd) RUNX2 levels and confirms specificity of the RUNX2 signal in MDA-MB-231 cells. **(F) **Western blot analyses of MDA-MB-231 cells that were first serum starved for 48 h and then treated with either 10% serum or 10% serum in the presence of the protein synthesis inhibitor cycloheximide (100 μM) for the indicated time periods (in hours). RUNX2 blots are represented as short (SE) or long (LE) exposures. Cycloheximide treatment results in a consistent decrease in RUNX2 levels together with a concomitant decrease in p21 and cyclin D1. Comparing RUNX2 protein levels at 8 h of serum activation with and without cycloheximide treatment reveals that RUNX2 is destabilized within 8 h after serum stimulation. These findings suggest that RUNX2 levels are tightly regulated by protein degradation during the cell cycle in MDA-MB-231 breast cancer cells as is the case in normal osteoblastic cells. Thus, elevated expression of RUNX2 protein in MDA-MB-231 breast cancer cells does not appear to be due to abrogation of a protein destabilizing mechanism.

RUNX2 is normally expressed in lineage-committed mesenchymal progenitor cells with a osteogenic cell fate, but RUNX2 expression is also aberrantly induced in different cancer cell types [8-17]. For example, RUNX2 is endogenously expressed in selected breast cancer cell lines as evidenced by detection of RUNX2 protein by western blot analysis in highly malignant MDA-MB-231 breast adenocarcinoma cells, but not in MCF7 breast adenocarcinoma cells that retain several characteristics of differentiated mammary epithelium (Figure 1A). Comparison of these two cell types may reveal the biological purpose of the aberrant ectopic expression of RUNX2 in non-osteoblastic cancer cells.

In normal osteoblasts, RUNX2 mRNA and protein levels are elevated in quiescent cells upon growth factor deprivation [23,41], but this growth-related regulation of *RUNX2 *gene expression is perturbed in several osteosarcoma cell types that express RUNX2 at elevated levels [23-26]. To assess whether expression of RUNX2 is regulated with respect to cell growth in MDA-MB-231 breast cancer cells, we performed serum starvation and stimulation experiments (Figure 1B). Cells were first cultured overnight in the presence of serum, and then cells were incubated with medium with or without serum for three days. Modest MDA-MB-231 cell proliferation is observed in cells maintained in normal serum (that is, cells increase in number but do not quite double even after three days), but cell proliferation is inhibited in cells subjected to prolonged serum starvation that enter a quiescent state (Figure 1B). Similar to MC3T3 osteoblasts [23,41], expression of RUNX2 increases with serum starvation and occurs concomitant with loss of phosphorylated Erk (Figure 1C). This finding suggests that RUNX2 expression is inversely correlated with cell proliferation and mitogen-dependent activation of the MEK-Erk pathway.

To examine whether expression of RUNX2 is directly controlled by mitogenic signaling pathways, we subjected proliferating MDA-MB-231 cells to distinct proliferation-associated kinase inhibitors and monitored RUNX2 protein levels by western blotting. MAPK signaling is known to regulate RUNX2 activity in different cell types [53,54]. The epidermal growth factor (EGF) receptor gene (EGFR/HER1/ERBB1) and other EGF receptors (HER2/NEU/ERBB2, HER3 and HER4) are frequently expressed in human breast cancer cells, including MDA-MB-231 cells [55,56]. Expression of EGFR and HER2 is associated with aggressive cancers and inhibitors of EGFR-HER signaling are tested in clinical trials [57]. Therefore, it is of interest to investigate links between EGFR signaling and RUNX2 expression. The results clearly show that RUNX2 protein is elevated by inhibition of the Erk pathway using the chemical inhibitor PD98059 or the EGFR pathway using AG494 (Figure 1D). However, inhibition of phosphoinositide 3-kinases (PI3Ks) by Wortmannin has no effect on RUNX2 protein levels (Figure 1D). Hence, inhibition of MAPK and EGFR signaling pathways attenuates RUNX2 levels in MDA-MB-231 cells.

Induction of cell proliferation in quiescent osteoblasts results in down regulation of *RUNX2 *gene expression at the level of transcription, as well as mRNA and protein accumulation [23,41]. Therefore, we tested whether serum stimulation of MDA-MB-231 cells can acutely regulate RUNX2 protein levels (Figure 1E). Parallel samples were treated with RUNX2 siRNA which establish the identity of the RUNX2 band in SDS-PAGE. Western blot results clearly show that RUNX2 levels remain high for at least four hours after serum stimulation and perhaps may be modestly and transiently upregulated around 1.5 hours after induction of cell growth as cells presumably exit from quiescence (G0) and enter the G1 phase (Figure 1E). These data suggest that RUNX2 levels are not acutely coupled to mitogenic signaling but may be down-regulated at a later proliferative stage (for example, the G1/S phase transition) that is beyond the four-hours-time point we have examined (Figure 1E).

To assess whether expression of RUNX2 in MDA-MB-231 cells is facilitated by protein stabilization, we reactivated proliferation in serum deprived MDA-MB-231 cells in the absence or presence of the protein synthesis inhibitor cycloheximide (100 μM). Assessment of the natural decay of RUNX2 over an eight-hour time course reveals that RUNX2 levels decrease with a half-life of about four-hours (Figure 1F), which is comparable to the half-life of exogenously expressed RUNX2 in COS cells [47]. For comparison, levels of the labile protein cyclin D1 are rapidly destabilized (less than two hours) albeit that levels rebound as the efficacy of protein inhibition by cycloheximide diminishes over time (Figure 1F). These data suggest that induction of RUNX2 protein expression in MDA-MB-231 cells is not due to abrogation of protein destabilizing mechanisms that normally degrade RUNX2, but may occur via other upstream gene regulatory mechanisms involving, for example, transcription factors or microRNAs.

### RUNX2 levels are inversely linked to Erk signaling

**Figure 2:**
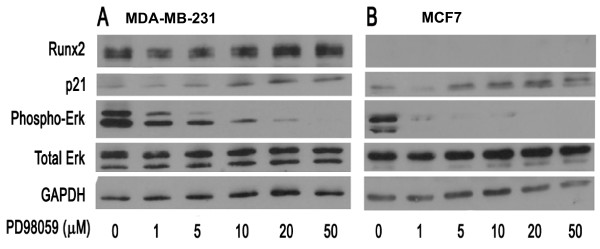
**Inhibition of ERK1/2 phosphorylation increases RUNX2 protein levels in MDA-MB-231 cells**. The coupling between mitogenic signaling pathways and RUNX2 protein expression was examined in MDA-MB-231 (**A**) or MCF7 cells (**B**). Each cell type was treated with the MEK1 inhibitor PD98059 for 2 h at the indicated concentrations (in μM). The protein levels of RUNX2, phospho-ERK, total ERK1/2, p21 and GAPDH were analyzed by western blotting. RUNX2 protein levels remained below the level of detection in MCF7 cells (**B**, top panel).

Because RUNX2 levels in MDA-MB-231 cells increase by serum deprivation and because Erk kinase phosphorylation is a key step in the mitogenic actions of serum-derived growth factors, we assessed whether Erk phosphorylation by MEK is biologically linked to increased RUNX2 protein expression by treating breast cancer cells with the MEK inhibitor PD98059 (Figure 2A, B). We performed western blot analysis of protein lysates from PD98059 treated MDA-MB-231 and MCF-7 cells to assess whether MEK inhibition can modulate RUNX2 expression in cells that ectopically express RUNX2 (that is, MDA-MB-231 cells) or cells in which RUNX2 is below the level of detection (that is, MCF-7 cells). The results show that Erk phosphorylation is decreased in a dose-dependent manner in both cell types (Figure 2). In each case, there is nearly a complete loss of phosphorylated Erk at 10 μM PD98059, albeit that MCF-7 cells appear to be considerably more sensitive to PD98059 inhibition (Figure 2B). Total levels of Erk are not appreciably altered in either MDA-MB-231 or MCF-7 cells at the doses of PD98059 we tested (Figure 2A, B). The concomitant inhibition of cell proliferation that arises from Erk inhibition is reflected by a modest increase in the levels of the CDK inhibitor p21/CDKN1A (Figure 2A, B). Importantly, the levels of RUNX2 are clearly upregulated in MDA-MB-231 cells upon treatment with PD98059 concentrations above 10 μM (Figure 2A), but RUNX2 levels remain below the level of detection in MCF7 cells (Figure 2B). Taken together, these results establish that RUNX2 protein levels are enhanced upon inhibition of mitogenic MEK-Erk signaling in MDA-MB-231 cells, but that this inhibition does not induce RUNX2 in MCF7 cells.

### RNAi mediated depletion of RUNX2 increases proliferation markers in but only modestly affects MDA-MB-231 cell number

**Figure 3:**
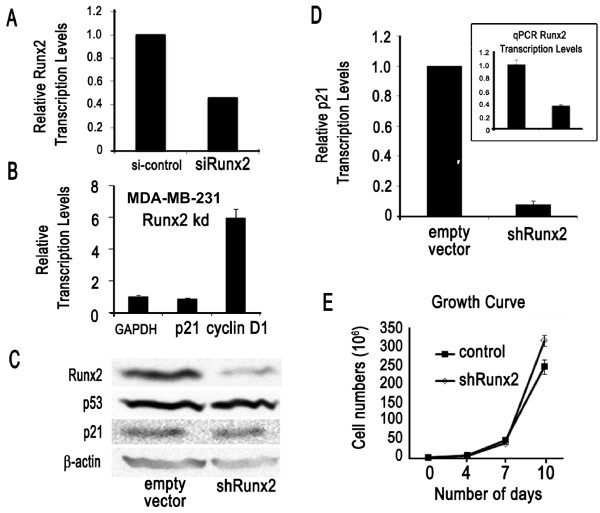
**Depletion of RUNX2 has a modest mitogenic effect in MDA-MB-231 cells**. **(A) **RUNX2 transcript levels in MDA-MB-231 cells, which were either treated with siRUNX2 or control non-silencing RNA (si-control), were measured using qRT-PCR analysis and normalized with GAPDH. **(B) **RUNX2 knockdown MDA-MB-231 cells results in increased expression of cyclin D1 and marginally decreased expression of p21 relative to GAPDH as measured using qRT-PCR analysis. **(C, D) **Protein levels detected by western blotting (**C**) and mRNA expression by qRT-PCR (**D**) was examined in MDA-MB-231 derived cell lines that were transduced with either shRUNX2 or empty vector control retroviruses. RNA was harvested from cells selected with puromycin (1 μg/ml) for two weeks. ShRNA mediated knock-down of RUNX2 mRNA (inset) dramatically reduced p21 mRNA levels (**D**), but putative compensatory changes may have prevented decreased accumulation of p21 protein (**C**). **(E) **Cell proliferation in MDA-MB-231 cells that either express shRUNX2 or contain empty vector (control) was measured by cell counting. Loss of RUNX2 has a modest positive effect on cell growth.

To assess the biological role of RUNX2 in MDA-MB-231 cells, we performed transient knockdown of RUNX2 with siRUNX2. Depletion of RUNX2 decreases the mRNA levels of the cell cycle inhibitor p21 and increases cyclin D1 mRNA levels indicating that loss of RUNX2 provokes a pro-proliferative response (Figure 3A, B). Constitutive knockdown of RUNX2 using a MDA-MB-231 cell line containing a retrovirus that expresses shRUNX2 RNA, also results in reduced p21 mRNA levels (albeit no commensurate change in p21 protein levels) (Figure 3C, D). Levels of p53 remain constant in MDA-MB-231 cells, which express a mutant p53 protein (R175H) which renders p21 gene transcription insensitive to p53. Reduction of RUNX2 has at best a marginal positive effect on cell growth over a period of 10 to 20 days (Figure 3E and data not shown). The rather modest observed variation in cell number at Day 10 may reflect modulations in cell cycle kinetics (duration of progression through each of the cell cycle stages), as well as the balance between cell survival and cell death. However, clear biological differences before Day 10, when shRNA treatments typically have more pronounced direct effects than at later stages, are not evident. Because loss of RUNX2 does not have major proliferative effects, it appears that RUNX2 activity is not critical for growth of MDA-MB-231 cells.

### RUNX2 expression in breast cancer cells promotes cell motility

**Figure 4:**
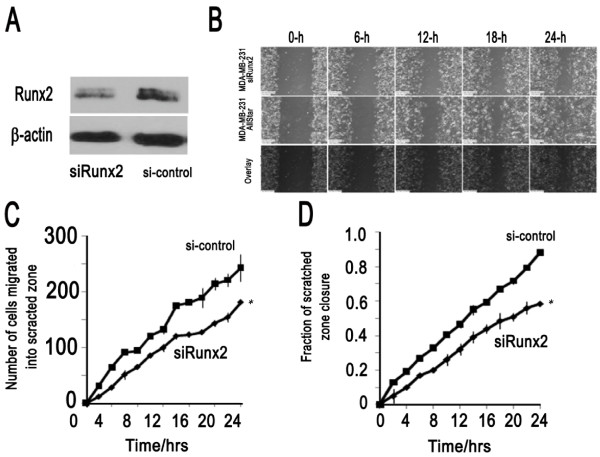
**RUNX2 silencing reduces migration of metastatic MDA-MB-231 breast cancer cells**. **(A) **Diminished expression of RUNX2 protein levels was observed by western blotting in lysates from MDA-MB-231 treated with siRUNX2. (**B**) Wound healing assay of MDA-MB-231 cells treated with siRUNX2 or a non-silencing RNA (AllStar Control) at five different time points. **(C) **Cell counts were performed to determine the number of cells migrating to the scratch zone after 24 h for MDA-MB-231 cells treated with siRUNX2 (*n *= 180 cells) or non-silencing AllStar Control (*n *= 242 cells). **(D) **Total percent closure was determined as a time-course after initiating the scratch. Control MDA-MB-231 cells transfected with AllStar Control (88%) achieved 29% percent closure, while cells treated with siRUNX2 (59%) exhibited reduced wound closure. Asterisk indicates statistical significance between siRUNX2 and control treatment (*P *< 0.01 based on ANCOVA test). Error bars represent standard deviation.

**Figure 5:**
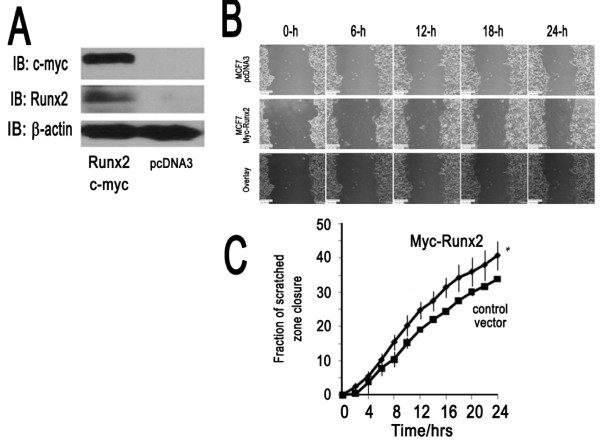
**RUNX2 expression increases cell migration in non-metastatic MCF7 breast cancer cells**. **(A) **Western blot analysis using a RUNX2 antibody and/or c-Myc epitope tag in MCF7 cells transfected with a Myc-RUNX2 expression vector. Levels of β-actin were used as a control for protein loading) **(B) **Images from a wound healing assay with MCF7 cells transfected with empty expression vector (pcDNA3 control) or a plasmid expressing Myc-RUNX2 at various time points (in hours). **(C) **Quantification of total percent closure of control MCF7 cells (pcDNA3; 34%) and Myc-RUNX2 expressing MCF7 cells (41%) revealed that RUNX2 expressing increases cell motility. Asterisk indicates statistical significance between Myc-RUNX2 and control treatment (*P *< 0.05 based on ANCOVA test). Error bars represent standard deviation.

Our preceding results indicate that RUNX2 is a potential cell growth inhibitor thus raising the question why aggressive human derived breast cancer cells would tolerate induction of *RUNX2 *gene expression. We postulated that RUNX2 may have a pathological side that increases the aggressiveness of MDA-MB-231 cells during metastasis. Therefore, we performed migration assays using a live imaging system and found that loss of RUNX2 by shRNA (Figure 4A) decreases cell motility in wound healing ('scratch') assays (Figure 4B). Time course results revealed that the number of cells migrating into the scratched area was significantly lower at both earlier (4 h) and later (24 h) time-points in cells depleted for RUNX2. We next investigated whether elevated expression of wild type RUNX2 in MCF7 cells, which are breast cancer cells with limited migratory potential and do not express detectable RUNX2 protein, would alter cell migratory potential. Indeed forced expression of RUNX2 increased motility in wound healing assays (Figure 5). Taken together, the results with MDA-MB-231 and MCF7 cells demonstrate that RUNX2 stimulates cell motility.

## Discussion

A number of recent studies have indicated that the osteogenic transcription factor RUNX2, is a suppressor of osteoblast growth, is frequently and aberrantly expressed in non-osseous cancer cells. Here, we show that expression of RUNX2 expression in MDA-MB-231 breast adenocarcinoma cells is reciprocally linked to mitogen-dependent enhancement of the MEK-Erk signaling pathway. However, unlike its normal activity in osteoblasts and osteoprogenitor cells [23,41,43], RUNX2 levels do not appear to be critically linked to cell proliferation in MDA-MB-231 breast cancer cells. Rather, our results indicate that RUNX2 levels are functionally coupled to cell motility in MDA-MB-231 or when introduced into MCF7 breast adenocarcinoma cells. Consistent with the 'wound healing' assays presented here, studies using Boyden chambers have shown that RUNX2 is required for both cell migration and invasion through Matrigel in prostate cancer cells [21]. It remains to be established whether modest quantitative effects on cell migration are directly relevant to metastatic disease. However, our current findings are certainly consistent with prior work showing that RUNX2 may promote the metastatic potential of breast cancer cells by modulating invasiveness and osteolytic properties [7-16]. Currently ongoing studies that address the biochemical basis for the relationships among RUNX2, cell migration and metastatic disease suggest that RUNX2 may regulate the expression of a distinct set of genes required for cell motility and adhesion (unpublished observations).

RUNX2 is most prominently expressed in osseous tissues during skeletal development, and normal RUNX2 function and levels are critical for normal growth and differentiation of osteoblasts [36-40]. RUNX2 is also expressed in non-osseous tissues including mesenchymal chondrocytes, vascular endothelial cells and breast epithelial cells at specific stages of development [54,58-65]. RUNX2 levels are deregulated in osteosarcoma [23-31] and chondrosarcoma cells [66-69]. Because RUNX2 acts as cell growth suppressor in osteosarcoma cells, the elevated or ectopic expression of RUNX2 that is observed in a diverse range of tumor cells of either osseous or non-osseous origin is rather enigmatic. Strikingly, there are no reports of RUNX2 point mutations in cancer and most biological associations between RUNX2 and cancers indicate gain-of-function effects, as is exemplified by over-expression of RUNX2 by protein stabilization or gene amplification in osteosarcoma [23-27] or ectopic induction by retroviral insertion in c-Myc related T cell lymphomas [32-35]. One emerging view that clarifies the paradoxical role of RUNX2 in cancer cells is that this factor may promote tumorigenesis by enhancing the expression of genes linked to metastatic properties (for example, cell motility and invasion) and/or angiogenesis once cells have succeeded in bypassing RUNX2 dependent growth restrictions [5-7,11-15,20-22,24-27,31,61,66]. Our previous studies have shown that RUNX2 expression is positively linked to estrogen receptor status in tissue biopsies of Stage II breast cancer patients [17]. This correlation is restricted to Stage II because loss of the estrogen receptor and gain of RUNX2 function is typical in highly aggressive breast cancer cells.

## Conclusions

The work presented here, which shows that RUNX2 stimulates cell motility of breast adenocarcinoma cells, corroborates the concept that RUNX2 represents a prognostic marker for breast cancer progression that is mechanistically linked to the metastatic potential of the cells in which it is expressed.

## Abbreviations

BSA: bovine serum albumin; CT: threshold cycles; DMEM: Dulbecco's modified Eagle's medium; DMSO: dimethyl sulfoxide; FBS: fetal bovine serum; GAPDH: glyceraldehyde-phosphate Idehydrogenase; HRP: horseradish peroxidase; PI3KS: phosphoinositide 3-kinases; PBS: phosphate-buffered saline; RUNX: Runt-related transcription factor; SDS: sodium dodecyl sulfate.

## Competing interests

The authors declare that they have no competing interests.

## Authors' contributions

DTL, GSS, MST, SMC and AJVW made substantial contributions to the conception, design and analysis of the experiments, to the interpretation of data, as well as to drafting and revising the manuscript. SSN, JBL, YI and PMV made substantial contributions to the interpretation of data, as well as to revising the manuscript. JL, XG, JP, BPP and HSK designed and executed experiments, as well as assisted in the drafting of the manuscript. All authors read and approved the final manuscript.
